# Spatial characteristics of non-communicable diseases and their associations to social conditions in a large urban cohort in Germany—Results from the Hamburg City Health Study

**DOI:** 10.1371/journal.pone.0301475

**Published:** 2024-04-09

**Authors:** Valerie Andrees, Ramona Bei der Kellen, Matthias Augustin, Jürgen Gallinat, Volker Harth, Hanno Hoven, Simone Kühn, Anne Lautenbach, Christina Magnussen, Nicole Mohr, Raphael Twerenbold, Ines Schäfer, Benjamin Waschki, Birgit-Christiane Zyriax, Jobst Augustin

**Affiliations:** 1 Institute for Health Service Research in Dermatology and Nursing (IVDP), University Medical Center Hamburg-Eppendorf (UKE), Hamburg, Germany; 2 Epidemiological Study Center, Hamburg City Health Study, University Medical Center Hamburg-Eppendorf (UKE), Hamburg, Germany; 3 Department of Psychiatry and Psychotherapy, University Medical Center Hamburg-Eppendorf (UKE), Hamburg, Germany; 4 Institute for Occupational and Maritime Medicine (ZfAM), University Medical Center Hamburg-Eppendorf (UKE), Hamburg, Germany; 5 Lise Meitner Group for Environmental Neuroscience, Max Planck Institute for Human Development, Berlin, Germany; 6 Department Endocrinology, Diabetology, Obesity and Lipids, University Medical Center Hamburg-Eppendorf (UKE), Hamburg, Germany; 7 University Heart and Vascular Center Hamburg, University Medical Center Hamburg-Eppendorf (UKE), Hamburg, Germany; 8 Center for Population Health Innovation (POINT), University Heart and Vascular Center Hamburg, University Medical Center Hamburg-Eppendorf (UKE), Hamburg, Germany; 9 German Centre for Cardiovascular Research (DZHK), Hamburg, Kiel, Luebeck, Germany; 10 Department of Pneumology, Hospital Itzehoe, Itzehoe, Germany; 11 Airway Research Center North (ARCN), German Center for Lung Research (DZL), LungenClinic Grosshansdorf, Großhansdorf, Germany; 12 Midwifery Science – Health Service Research and Prevention, Institute for Health Services Research in Dermatology and Nursing (IVDP), University Medical Center Hamburg-Eppendorf (UKE), Hamburg, Germany; Jimma University, ETHIOPIA

## Abstract

**Background:**

Non-communicable diseases (NCDs) are responsible for many deaths. They are associated with several modifiable and metabolic risk factors and are therefore prone to significant regional variations on different scales. However, only few intra-urban studies examined spatial variation in NCDs and its association with social circumstances, especially in Germany. Thus, the present study aimed to identify associations of personal risk factors and local social conditions with NCDs in a large German city.

**Methods:**

This study is based on a population-based cohort of the Hamburg City Health Study including 10,000 probands. Six NCDs were analyzed (chronic obstructive pulmonary disease [COPD], coronary heart disease [CHD], diabetes mellitus, heart failure, depression, and hypertension) in 68 city district clusters. As risk factors, we considered socio-demographic variables (age, sex, education) and risk behaviour variables (smoking, alcohol consumption). Logistic regression analyses identified associations between the district clusters and the prevalence rates for each NCD. Regional variation was detected by Gini coefficients and spatial cluster analyses. Local social condition indexes were correlated with prevalence rates of NCDs on city district level and hot-spot analyses were performed for significant high or low values.

**Results:**

The analyses included 7,308 participants with a mean age of 63.1 years (51.5% female). The prevalence of hypertension (67.6%) was the highest. Risk factor associations were identified between smoking, alcohol consumption and education and the prevalence of NCDs (hypertension, diabetes, and COPD). Significant regional variations were detected and persisted after adjusting for personal risk factors. Correlations for prevalence rates with the local social conditions were significant for hypertension (r = 0.294, p < 0.02), diabetes (r = 0.259, p = 0.03), and COPD (r = 0.360, p < 0.01).

**Conclusions:**

The study shows that regional differences in NCD prevalence persist even after adjusting for personal risk factors. This highlights the central role of both personal socio-economic status and behaviors such as alcohol and tobacco consumption. It also highlights the importance of other potential regional factors (e.g. the environment) in shaping NCD prevalence. This knowledge helps policy- and decision-makers to develop intervention strategies.

## Introduction

Non-communicable diseases (NCDs) such as ischemic heart disease, different forms of cancer, chronic obstructive pulmonary disease (COPD) and diabetes mellitus are responsible for 41 million deaths each year, equivalent to 74% of all deaths globally [1]. These diseases are characterized by their complexity and prolonged duration, often spanning several decades. They are typically associated with genetic, physiological, environmental, socioeconomic and behavioural risk factors [1].

Several modifiable behavioural risk factors (e.g., tobacco use, the harmful use of alcohol, physical inactivity and unhealthy diet) and metabolic risk factors (e.g., raised blood pressure, overweight/obesity) additionally increase the risk of NCDs [1]. In high-income countries, socioeconomic status (SES) is known to be strongly associated with NCDs, especially in urban populations [2, 3]. People in disadvantaged social situations are more likely to engage in the use of harmful substances such as tobacco and alcohol. They are also more likely to exhibit unhealthy dietary behaviour, a lack of physical activity and limited access to health services [4, 5]. Furthermore, oftentimes their financial constraints confine them to live in low-quality residential areas with a high level of noise, etc. In addition, depending on their level of education, they tend to lack the necessary knowledge to positively influence their health behaviour or choices [6]. The risk factors that precipitate NCDs are often not immediately related to how individuals live their daily lives in terms of timing. That is, the negative health consequences of unhealthy lifestyles may take years to manifest. As they struggle with the day-to-day issues, challenging social situations, and stress related to SES, many people pay little attention to preventing future NCDs or treating existing NCDs [7]. This oversight significantly contributes to an increased risk of NCDs in individuals in difficult social circumstances.

Depending on these risk factors and the regional composition of the population, spatial differences in the prevalence of NCDs can emerge. Many studies have shown regional inequalities in population health at national level, particularly with regard to NCDs: e.g., coronary heart disease (CHD) [8], hypertension [9, 10], diabetes [11, 12], cancer [13], chronic obstructive pulmonary disease [14], or depression [15, 16]. However, the study of regional differences in NCDs below the national level is often limited by the fact that relevant data on the determinants (especially on health behaviours, e.g., alcohol consumption, smoking) are inadequate (e.g., spatially only roughly aggregated) or not available at all. This makes it difficult to identify the causes of the regional prevalence of NCDs, especially regarding the social situation. In this respect, it is important to examine the spatial variation of NCDs and the importance of social location at the smallest possible spatial level. There are many studies of intra-urban differences in life expectancy and mortality [17, 18]. However, there are only a few small-scale or intra-urban studies examining the spatial variation in NCDs and their association with social circumstances.

Malta et al. (2014) [19] analysed the main risk factors for NCDs in nine urban districts of Belo Horizonte (Brazil). They found that neighbourhoods with low alcohol consumption, a high proportion of non-smokers, high levels of physical activity and a healthy diet (in this case, consumption of fruits and vegetables) were also associated with fewer NCDs in the population. Tumas et al. (2022) [20] examined the relationship between education and the occurrence of selected NCDs (diabetes, hypertension) and risk factors (overeating, smoking, binge drinking) in different Argentinean cities. Overall, the results show the importance of education respectively that a low level of education is associated with a higher risk of NCDs. This association is not only evident when comparing cities, but also within cities.

Landy et al. (2012) [21] examined the adverse health profile of Glasgow for selected NCDs (e.g. depression, anxiety, heart attack, COPD) compared to the rest of Scotland through differences in socio-economic (including income, relationship status), behavioural (including alcohol consumption, tobacco consumption, diet) and biological (including obesity, blood pressure) risk factors. Based on the outcome of the study, which was a representation of the majority, a conclusion can be drawn that the differences between Glasgow and the rest of Scotland can be explained by a combination of area and individual socio-economic circumstances. However, no associations were found for anxiety, heart attack, and obesity.

In Germany, there are limited studies [22] examining the associations between NCDs and social situation at a small scale and within cities. This is probably due to a lack of suitable data. The Hamburg City Health Study (HCHS) provides such a dataset for a typical large German city. In the present study, we aimed to answer the following research questions:

1. What are the spatial characteristics of the most important NCDs in a typical German large city?
2. How are personal risk factors associated with these NCDs in a typical German large city?
3. How are the local social conditions in a typical German large city associated with the risk of these NCDs?

## Materials and methods

### Study population

The present dataset is a cross-sectional social-epidemiological cohort study from the Hamburg City Health Study (HCHS, clinicaltrials.gov: NCT03934957). The rationale and design of HCHS has already been published [23]. All research conducted in the trial conforms to the ethical standards of the Institutional and National Research Committee and was conducted in accordance with the Declaration of Helsinki of 1964 and its subsequent amendments. The HCHS trial is registered on ClinicalTrial.gov (NCT03934957). It has been approved by the local ethics committee of the Hamburg Medical Association (PV5131). In addition, the study was approved by the data protection officer of the University Medical Center Hamburg-Eppendorf (UKE) and the data protection officer of the City of Hamburg. HCHS is an ongoing population-based cohort study with a targeted random sample of 45,000 residents of Hamburg (Germany) between 45 and 74 years at inclusion to identify risk factors of major chronic diseases. Start of the survey (10,000 cohort) was 01 February 2016, end at 07 November 2018. The HCHS Study Centre provided the data to the authors for analysis on 27 March 2022. Data collection in HCHS is performed in different ways. First, participants complete comprehensive online-questionnaires. These questionnaires comprise questions on environmental conditions, health-related behaviours, quality of life, medical history, and health care utilization. The extensive clinical examination-program takes place at the study centre during the baseline visit. During this visit, the participants take part in various validated examinations assessing more than 30 chronic and acute diseases. Participants were informed in advance about the study, data collection and use, and gave their written consent. The authors had no information during or after the survey that could have identified the participants.

### Data and diagnostics

**Fig 1:**
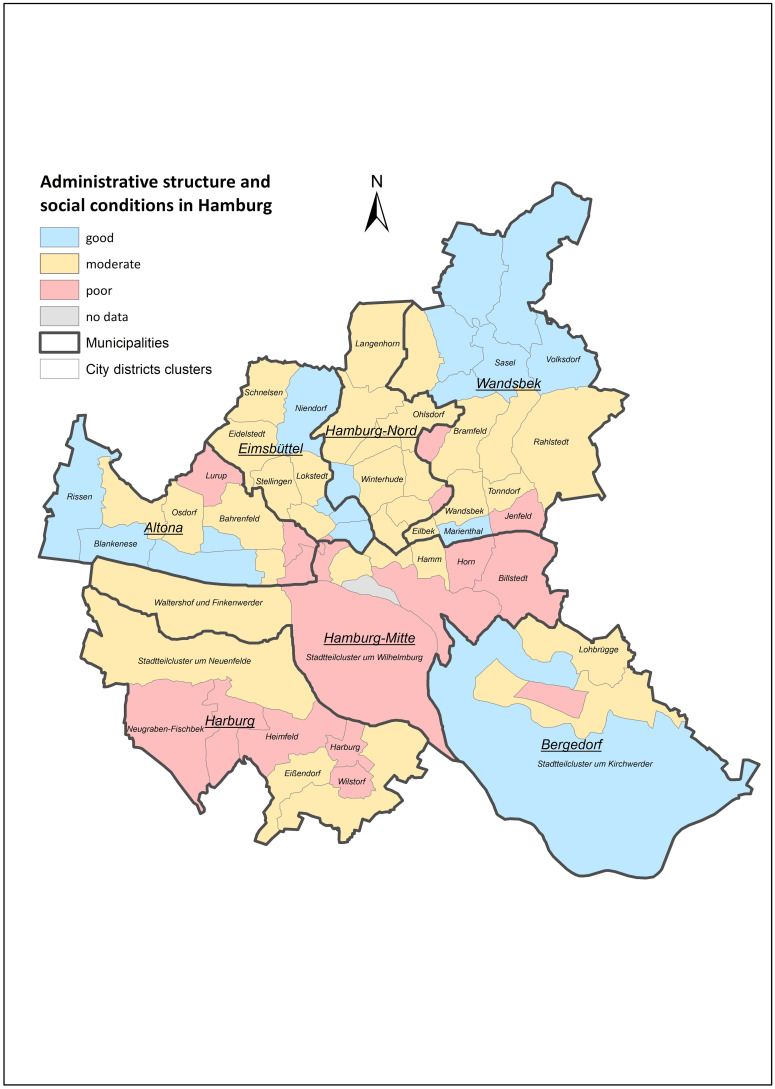
Administrative structure and local social conditions in Hamburg.

This study is based on a cohort of the first 10,000 participants with validated baseline data. The spatial variable was defined as the place of residence (zip-code) of the participants. Included in the study were persons who have lived in their current place of residence for at least ten years to assume a residential effect on NCDs. The city of Hamburg is structured in 104 districts and seven municipalities (administrative structure). To achieve sufficient sample sizes per district, some districts were summarised into clusters with at least 3,000 inhabitants by Erhart et al. (2013) [22], resulting in 68 analysed district clusters in total (Fig 1).

We considered those NCDs with the largest impact on morbidity and mortality, as defined by the WHO [5]. We focused on the six most common diagnoses within the HCHS study population: COPD, CHD, diabetes mellitus (here we combined Type 1 and Type 2 diabetes), heart failure and hypertension. In addition, we chose depression as representative for mental disorders. Hypertension takes on a special role here, as it can be seen as both a disease and a risk factor. Due to the high prevalence, we included hypertension as a NCD. Cancer was however not considered due to the low number of cases at the district cluster level.

Several personal risk factors were selected for further analysis. The selection was based on literature [3, 24] and on the model for classifying risk factors for NCDs [25]. We included socio-demographic variables (age, gender, education) and risk behaviour variables (smoking, alcohol consumption). Educational status was defined according to the International Standard Classification of Education (ISCED) [26] (Table 1). For the regional social conditions in those district clusters, we applied a score developed by Erhart et al. (2013) [22]. The score is based on a principal component analysis and takes into account 26 variables (e.g. education, income). The factor scores were grouped into a three-level form: good, moderate, and poor social conditions (Fig 1).

**Table 1:** Overview of personal level data used in this study.

	Name	Description
**Diseases**	Chronic obstructive pulmonary disease (COPD)	Self-reported chronic bronchitis or COPD
	Coronary heart disease (CHD)	Self-reported coronary heart disease
	Depression	PHQ-9 questionnaire [27, 28]
		0 → No depression
		1–4 → Minimal depression
		5–9 → Mild depression
		10–14 → Moderate depression
		> 14 → Heavy depression
		(PHQ-9 > 9 defined as present depression in the study)
	Diabetes mellitus	Self-reported insulin-dependent or non-insulin-dependent diabetes or Glucose > 126 *mgdl* if it has been more than eight hours since the last meal or Glucose > 200 *mgdl* if it has not been more than eight hours since the last meal or taking medication of group A10 (ATC code)
	Heart failure	Self-reported heart failure or LVEF < 40% and presence of dyspnoea or edema or taking loop diuretics or aldosterone antagonists or LVEF ≥ 40% and NT-proBNP > 125 *ngl* and presence of dyspnoe or edema or taking loop diuretics or aldosterone antagonists
	Hypertension	Self-reported hypertension or taking medication of group C09A, C09C, C07A, C03C, C03A, C03D, C08C, C02D, C02A, C09X or C01D (ATC code) or systolic blood pressure of > 140 mmHg or diastolic blood pressure of > 90 mmHg
**Personal Risk Factors**	Age	Self-reported
	Gender	Self-reported
	Education	Categorised into low, middle, and high according to the International Standard Classification of Education (ISCED) [26]
	Smoking	Self-reported (yes/no)
	Alcohol consumption	People were documented as drinking if they reported drinking alcohol at least twice a week

### Statistical analyses

First, we analysed all variables descriptively with percentages for categorical variables and mean, median, and standard deviation for continuous variables. (Lifetime)-prevalence rates and their 95% confidence intervals for each examined disease were calculated. To examine associations between the individual district clusters with the prevalence rates, logistic regression analyses were conducted for each NCD separately: In each model, the NCD was used as a dependent variable. Independent variables were age, gender, education, smoking, and alcohol consumption and the individual district clusters.

To detect the presence and the extent of regional variations of the diseases’ prevalence rates, we calculated the Gini coefficient with the associated Lorenz curve and performed spatial cluster analyses of adjusted prevalence rates. The utilized prevalence rates were adjusted for gender, age, education, smoking and alcohol. The Gini coefficient is used in spatial epidemiology to express regional differences in health and health care [29]. It ranges from 0 to 1, with 1 being the highest possible inequality. The associated Lorenz curve graphically displays the deviation of the observed distribution from perfect equality.

In the next step, we correlated the regional social conditions with the prevalence rates of NCDs in the city district clusters with Pearson’s correlation coefficient. For diseases with a significant correlation (p < 0.05), we then identified spatial clusters with significantly high or low prevalence values. For this, we performed hot-spot analyses with Getis-Ord Gi* statistics ArcGIS 10.8.2 (ESRI Inc., Redlands, CA, USA). This method detects whether a rate in a district cluster is higher than expected, a hot-spot, or lower than expected, a cold-spot [30, 31]. A hot-spot is characterised by a positive z-score and a cold-spot by a negative z-score. This method takes neighbouring districts into account and spots become significant if neighbouring districts collectively have either high or low values. The hot-spots and cold-spots were detected with three different confidence levels (90%, 95%, 99%), dependent on z-scores and p-values. To illustrate, how much of the regional variations can be explained by the independent variables from the abovementioned logistic regression analyses, we performed the hot-spot analyses with three different prevalence rates per diagnosis: I. Raw prevalence rate, II. Age- and gender-adjusted prevalence rate, and III. Fully adjusted prevalence rates for all significant variables from regression analysis.

The analyses were performed with ArcGIS 10.8.2, QGIS 3.22.13-Białowieża (QGIS Development Team) and with R Core Team R (R Foundation for Statistical Computing, Vienna, Austria).

## Results

### Study population

Of the 10,000 cohort, data for 7,308 participants with a residence time of at least ten years were available. Overall, 92% of the participants have not relocated within the last ten years. Mean age was 63.1 ± 8.3 years, 51.5% were female. While age and gender were comparable across the seven municipalities, there were significant differences in education, smoking and alcohol consumption (Table 2).

**Table 2:** Baseline characteristics of the study population, stratified by region/municipalities.

Municipalities	n	Age in years (mean [SD])	Female, n (%)	Education, n (%)			Smoker, n (%)	Alcohol, n (%)
				Low	Middle	High		
Total Hamburg	7,308	63.1 (8.3)	3,765 (51.5)	336 (4.7)	3,753 (52.1)	3,117 (43.3)	1,375 (18.9)	2,658 (46.9)
Altona	1,056	62.8 (8.3)	523 (49.5)	49 (4.7)	433 (41.4)	563 (53.9)	222 (21.1)	441 (53.4)
Bergedorf	546	62.6 (8.0)	287 (52.6)	34 (6.3)	318 (58.8)	189 (34.9)	83 (15.2)	151 (35.4)
Eimsbüttel	1,231	63.0 (8.5)	658 (53.5)	47 (3.9)	608 (50.3)	554 (45.8)	244 (19.9)	488 (49.8)
Hamburg Central	624	62.4 (8.2)	308 (49.4)	60 (9.8)	369 (60.4)	182 (29.8)	153 (24.6)	182 (40.8)
Hamburg North	1,166	62.9 (8.3)	605 (51.9)	32 (2.8)	584 (51.0)	530 (46.2)	248 (21.3)	453 (50.6)
Harburg	546	63.5 (8.3)	275 (50.4)	33 (6.1)	304 (56.4)	202 (37.5)	89 (16.4)	171 (40.8)
Wandsbek	2,139	63.5 (8.2)	1,109 (51.8)	81 (3.8)	1,137 (53.8)	897 (42.4)	336 (15.8)	772 (46.1)

SD: Standard deviation

### Prevalence of NCDs

The proportion of patients with hypertension was 67.6%, followed by diabetes (8.6%) and COPD with 7.0% (Table 3). The NCDs with the lowest proportions in the study population are heart failure (4.8%), CHD (5.2) and depression (6.1%).

**Table 3:** Lifetime prevalence of the analysed NCDs in Hamburg, N = 7,308.

Disease	n	%	95% CI
CHD	375	5.2	4.7–5.7
COPD	486	7.0	6.4–7.6
Depression	409	6.1	5.5–6.7
Diabetes	586	8.6	8.0–9.3
Heart failure	348	4.8	4.3–5.3
Hypertension	4,742	67.6	66.5–68.7

n: number of cases; 95% CI: 95% confidence interval; CHD: coronary heart disease; COPD: chronic obstructive pulmonary disease

### Risk factors and NCDs

Logistic regression analyses were used to quantify the association between the risk factors and the prevalence of NCDs (Table 4). Age was positively associated with all NCDs. Being female was a risk factor for COPD and depression and a protective factor for CHD, diabetes, heart failure and hypertension. The highest educational level (ISCED 3) was negatively associated with hypertension, diabetes, and depression. Smoking was positively associated with COPD and depression. Consumption of alcohol at least twice a week was less common for people with diabetes, heart failure, and depression.

**Table 4:** Excerpt results of the logistic regression analyses for the associations of risk factors with NCDs.

	COPD		CHD		Hypertension		Diabetes		Heart failure		Depression	
	OR (95% CI)	p	OR (95% CI)	p	OR (95% CI)	p	OR (95% CI)	p	OR (95% CI)	p	OR (95% CI)	p
**Age**	**1.0 (1.0–1.0)**	**< 0.001**	**1.1 (1.1–1.1)**	**< 0.001**	**1.1 (1.1–1.1)**	**< 0.001**	**1.1 (1.0–1.1)**	**< 0.001**	**1.1 (1.1–1.1)**	**< 0.001**	**1.0 (1.0–1.0)**	**< 0.001**
**Female gender**	**1.4 (1.1–1.8)**	**0.006**	**0.3 (0.2–0.4)**	**< 0.001**	**0.5 (0.4–0.6)**	**< 0.001**	**0.5 (0.4–0.6)**	**< 0.001**	**0.6 (0.5–0.8)**	**0.001**	**1.7 (1.3–2.2)**	**< 0.001**
**ISCED 2**	0.8 (0.5–1.3)	0.378	0.7 (0.4–1.4)	0.329	**0.7 (0.5–1.0)**	**0.043**	**0.7 (0.4–1.0)**	**0.068**	0.8 (0.5–1.4)	0.337	**0.5 (0.3–0.8)**	**0.001**
**ISCED 3**	0.6 (0.4–1.0)	0.04	0.6 (0.4–1.2)	0.127	**0.5 (0.4–0.7)**	**< 0.001**	**0.5 (0.3–0.8)**	**0.004**	0.7 (0.4–1.3)	0.208	**0.4 (0.3–0.7)**	**0.001**
**Smoking** *	**2.5 (1.9–3.2)**	**< 0.001**	1.0 (0.7–1.5)	0.812	**0.8 (0.7–1.0)**	**0.010**	1.1 (0.8–1.5)	0.517	1.2 (0.8–1.7)	0.297	**1.4 (1.1–1.9)**	**0.009**
**Alcohol consumption** **	0.8 (0.7–1.1)	0.156	0.9 (0.7–1.2)	0.478	1.1 (0.9–1.2)	0.373	**0.5 (0.4–0.6)**	**< 0.001**	**0.7 (0.6–1.0)**	**0.027**	**0.6 (0.5–0.8)**	**< 0.001**

COPD: chronic obstructive pulmonary disease; CHD: coronary heart disease; ISCED: International Standard Classification of Education; OR: odds ratio; 95% CI: 95% confidence interval; p: p-value;

*self-reported yes;

** self-reported yes at least twice a week

### Regional variation and NCD prevalence

**Fig 2:**
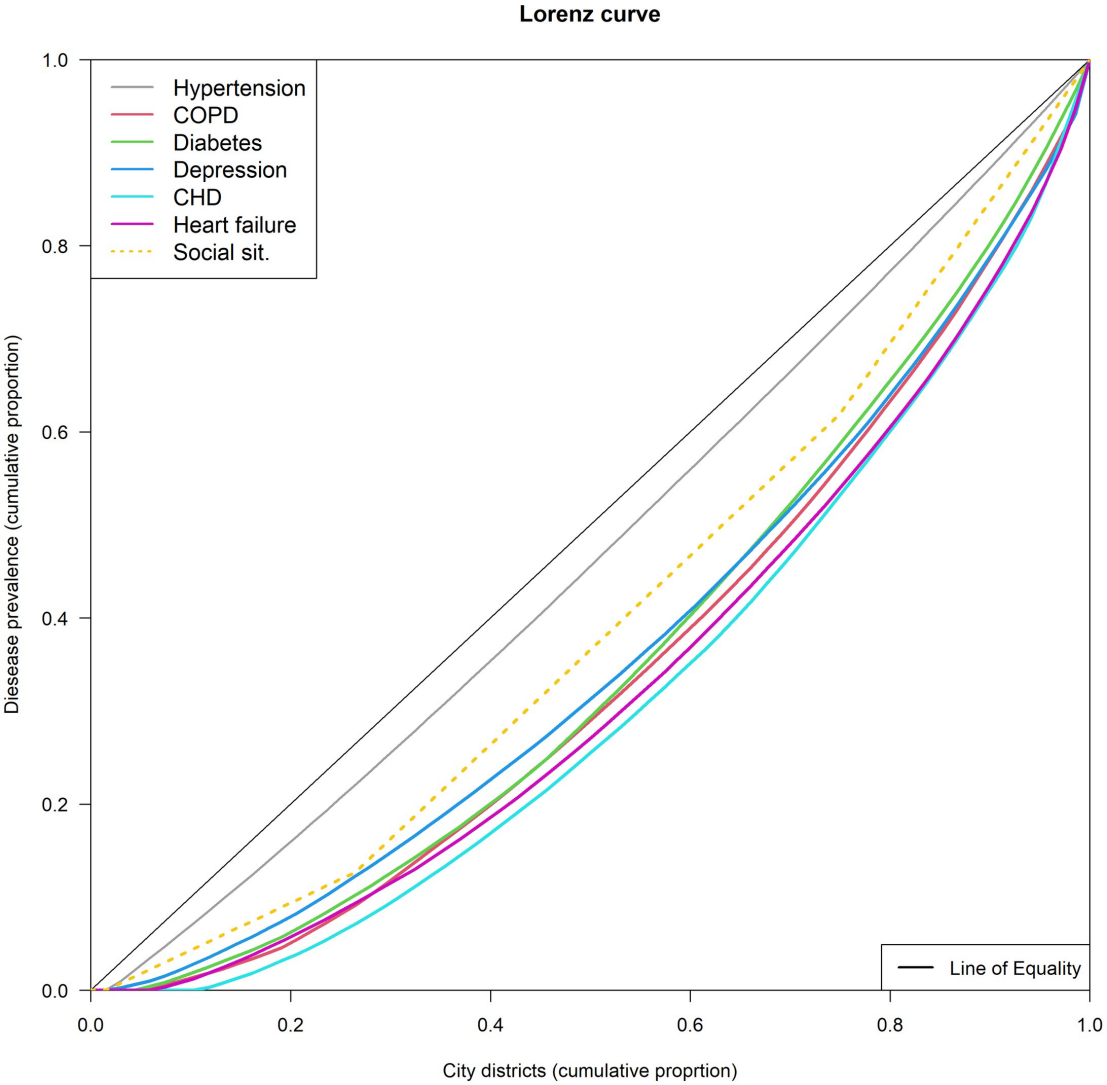
Lorenz curves and Gini coefficients for prevalence rates of non-communicable diseases in Hamburg after adjustment for age, gender, education, smoking, alcohol, and social conditions.

The extent of regional differences in prevalence rates in Hamburg is described with the Gini coefficient and the associated Lorenz curves (Fig 2). The strongest spatial variation is shown for CHD (Gini 0.36), followed by heart failure (0.34) and COPD (Gini 0.31). The smallest variation is found in hypertension (Gini 0.067), which can also be seen in the Lorenz curve. The curve is close to the line of equality. Furthermore, the inequality of the social conditions is apparent with a Gini coefficient of 0.20.

### Correlations with social conditions and hot-spot analyses

The Pearson’s correlation for NCD prevalence rates with the social conditions in the city district clusters were significant for three diseases: hypertension (r = 0.294, p < 0.02), diabetes (r = 0.259, p = 0.03), and COPD (r = 0.360, p < 0.01). For those diseases, we performed hot-spot analyses.

**Fig 3:**
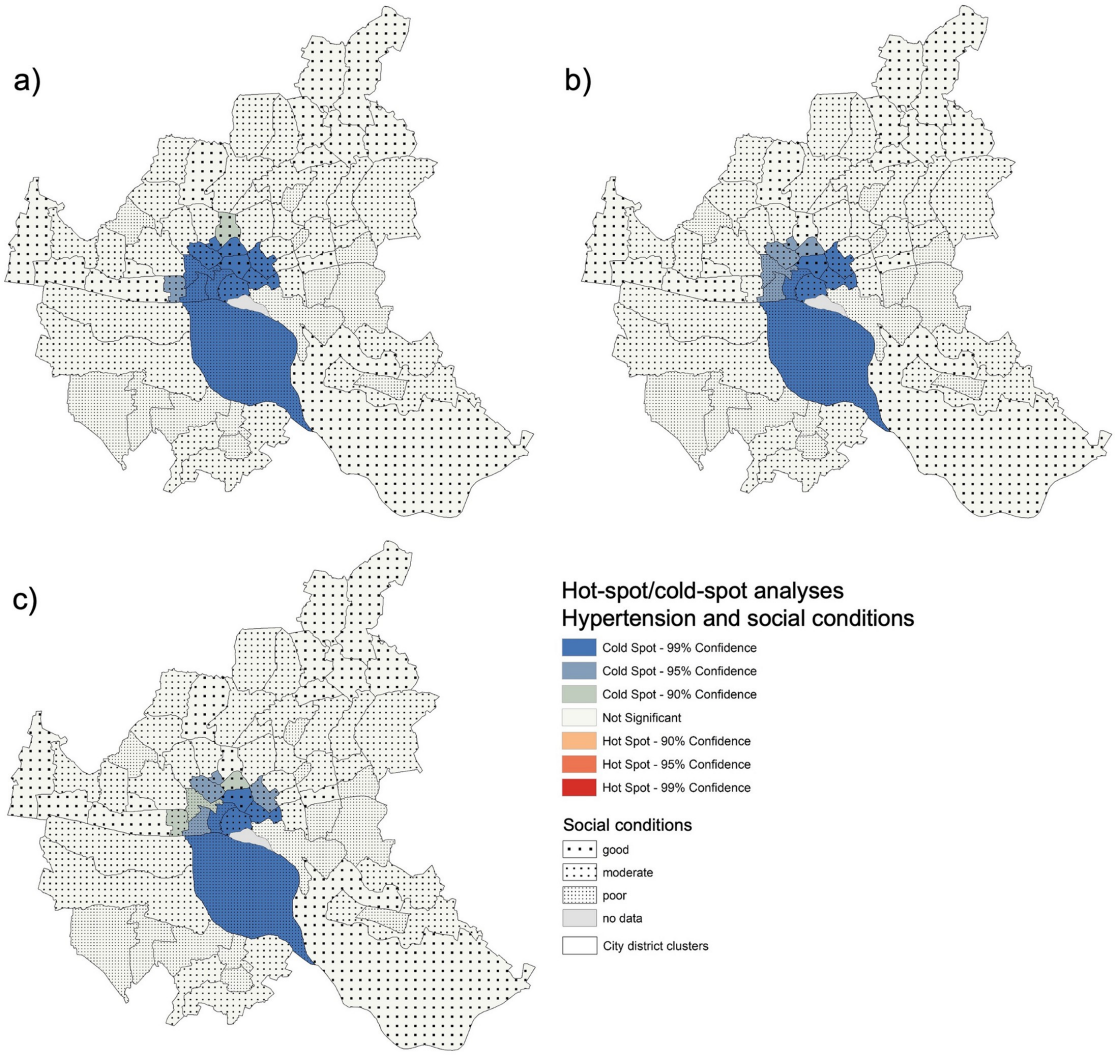
Social conditions, hot-spots, and cold-spots of hypertension prevalence rates in Hamburg. a) unadjusted, b) partially adjusted, c) fully adjusted.

Hypertension prevalence rates (Fig 3) reveal no hot-spot in Hamburg, but a large cold-spot cluster in the (southern) center of Hamburg. These districts predominantly present moderately to good social conditions. The adjustment for age- and gender (b) as well as the fully adjusted (c) rates resulted in truly little modification of the cold-spot. Most district clusters in the cold-spot remained significant with 99%-percent and some became less significant with 95% or 90% after adjustments.

**Fig 4:**
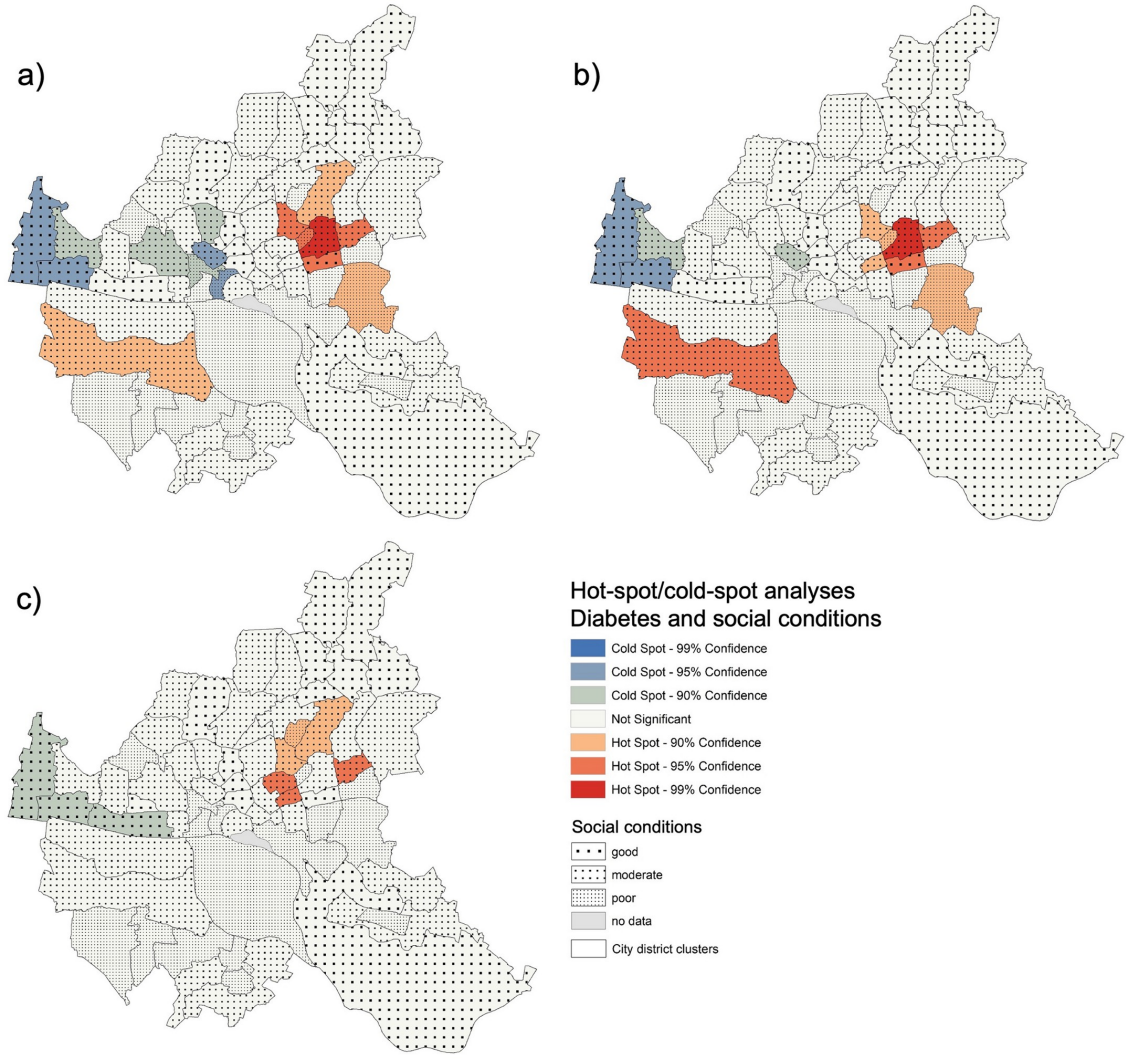
Social conditions, hot-spots, and cold-spots of diabetes prevalence rates in Hamburg. a) unadjusted, b) partially adjusted, c) fully adjusted.

The hot-spot analyses for diabetes (Fig 4) show significant hot-spots and low-spots in Hamburg. The non-adjusted prevalence rates (a) show a large hot-spot area in the east of Hamburg and a hot-spot in the south-west. These districts predominantly show moderate social conditions. In addition, there are cold spots in the middle of the city and in the west. These districts mostly present moderate to good social conditions. After adjustment for age and gender (b), the eastern hot-spot areas become smaller, and the western hot-spot shows a higher confidence level, whereas the cold-spot in the middle of the city is minimized. With full adjustment (c), the western hot-spot disappears, the eastern hot-spot becomes smaller with a lower confidence level and the western cold-spot confidence level decreases.

**Fig 5:**
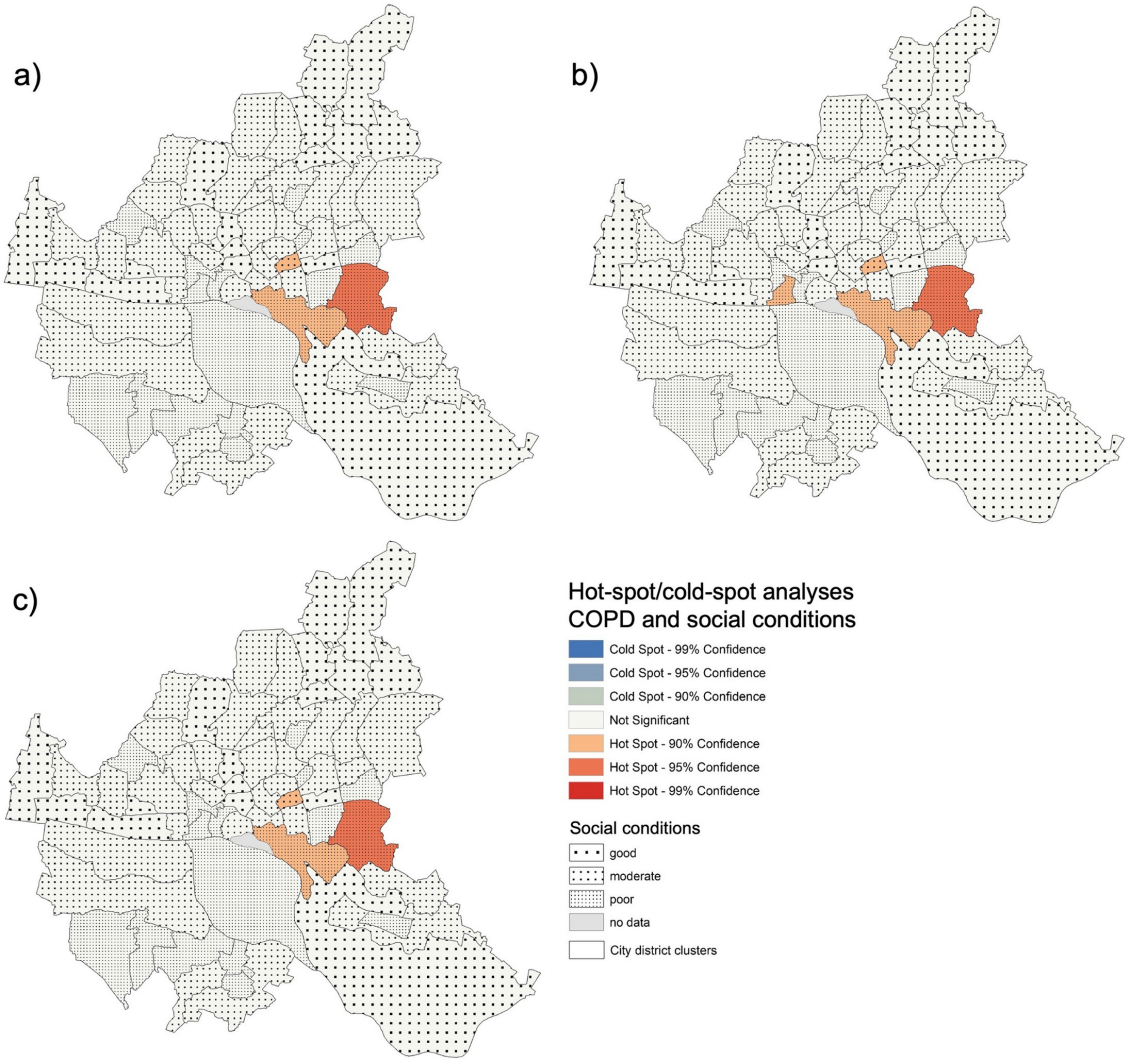
Social conditions, hot-spots, and cold-spots of COPD prevalence rates in Hamburg. a) unadjusted, b) partially adjusted, c) fully adjusted.

For COPD, no cold-spots were detected. Hot-spots were found in some urban districts in the east of Hamburg. Here, the districts show mostly poor social conditions, and one district shows moderate social conditions. This applies to both the non-adjusted and the (full) adjusted data (Fig 5).

## Discussion

The overall aim of this study was to determine the regional prevalence rates of relevant NCDs in the HCHS population, considering the local social conditions. There were significant regional differences in the prevalence of the selected NCDs. Additionally, within the study population striking regional differences in both educational level and in the consumption of alcohol and tobacco were found.

In particular, the prevalence of hypertension (67.6%) was significantly higher than that of other NCDs (e.g., diabetes 8.6%, COPD 7.0%). Presumably, the high prevalence of hypertension is due to the relatively high age (63.1 years) of the study population. Neuhauser et al. (2017) [10] also reported a prevalence (12-month prevalence rate) rate of 65.1% (≥ 65 years, male) in their study on hypertension prevalence in Germany. In contrast, Heidemann et al. (2017) [32] showed a significantly higher prevalence of diabetes (21.1%) (≥ 65 years, male, 12-month prevalence rate) compared to the present study. Similar results are also found for COPD: Steppuhn et al. (2017) [14] reported a prevalence of 12.5% (≥ 65 years, male, 12-month prevalence rate) which is also higher than in the present study. The difference (diabetes and COPD) in prevalence rates between this study and other studies is probably due to the different composition of the study populations. The comparative populations were based on representative population surveys in Germany, which also encompass rural areas.

In terms of risk factors, associations were identified between smoking, alcohol consumption and the education of the participants with the prevalence of NCDs (COPD, diabetes, hypertension). According to Tumas et al. (2022) [20], education, here operationalised as ISCED [26], can be considered as a proxy for socioeconomic status, as it is closely related to income. The prevalence of self-reported hypertension, diabetes and depression was associated with education: a lower educational level was associated with an increased risk of these diseases. For the other NCDs, there was no clear association with education. A positive association was found between COPD and smoking. This association was also reported by Wheaton et al. (2019) [33]. The association between smoking and depression remains unclear in the literature, as both positive and negative associations are discussed [34]. We also found negative associations for diabetes, heart failure and depression with alcohol consumption. For diabetes and alcohol, Polsky and Akturk (2017) [35] describe in their review that light to moderate alcohol consumption reduces the incidence of diabetes, whereas heavy drinkers and binge drinkers have an increased risk of diabetes. The results of our study cannot be directly compared with those of Polsky and Akturk or other studies. The reason for this lack of comparison with this study is that alcohol consumption in not further categorised into levels (low, moderate, or high consumption). As with diabetes, the relationship between heart failure and alcohol consumption is complex. Although it has been shown that former drinkers have a higher risk of heart failure than total abstainers, the results are inconclusive. Factors such as regularity, quantity, gender, and pre-existing conditions are important [36]. The relationship between alcohol use and depression is also complex, and the results here cannot be clearly related to the literature. Although it is estimated that up to 40% of people with depression have had a lifetime history of addiction or alcohol dependence [37], this does not provide a clear link.

Numerous studies have shown striking spatial variation of deprived populations within cities [38–40]. This includes the uneven spatial distribution of related risk factors, which can be reflected in the incidence of NCDs. This association was also observed here, using the example of a large German city. However, social inequality in the city does not always appear to have the same associations with different NCDs, as the analyses demonstrate. For example, hypertension seems to be the least affected by social inequality, whereas COPD is more affected. Erhart et al. (2013) [22] were also able to show this association, which varies depending on the disease, in their analysis of the intra-urban incidence of certain diseases. The authors describe those chronic diseases whose causes are strongly associated with lifestyle habits and social and environmental influences. In addition, they show a direct spatial association with the social situation. In contrast, acute causes of treatment in children, for example, showed little measurable relationship with socio-regional location.

In this study, significant regional differences persisted even after adjusting for personal risk factors. This implies that the variables in the models did not sufficiently explain all the variation. After adjusting for our risk factors, we found that the social situation remained strongly correlated with the local prevalence of three major diseases: hypertension, diabetes, and COPD. This confirms that not only personal SES and behaviour (in this case, alcohol, and tobacco consumption), but also other regional environmental factors may play an important role. They may alter environmental conditions [41, 42] such as air quality, noise, access to healthcare and crime rates.

One of the strengths of this study is the database, which allows statements about the relationship between NCDs and the social situation from a small spatial perspective. It is worth noting that 92% of the people who participated in the study resided in the same place for ten years or more. This high percentage suggests a high validity of the results and, ultimately, how people’s quality of life and health is influenced by their choice of place of residence.

However, as we used lifetime prevalence rates for the analyses, the exact time of disease onset is unclear, so ultimately no temporal sequences can be inferred. Another limitation is that a cross-sectional study design only allows us to detect the presence of associations, not their direction. For education and social situation, it is likely that lower SES and less knowledge about healthy lifestyles led to poorer health literacy and health behaviour, e.g., regarding dietary behaviour, physical activity, and prevention [43, 44]. Interestingly, we found that participants with hypertension were less likely to smoke, and people with diabetes, heart failure and depression were less likely to consume alcohol. The assumption is that they improved their health behaviour after being diagnosed with the condition. However, we were not able to verify the latter in the study, as no longitudinal data were available. Nor can we completely rule out the possibility of misclassification. The information on alcohol consumption should also be interpreted with caution. Social desirability may have played a role, so consumption may have been underestimated. Another limitation of the study is that COPD and CHD were self-reported by the patients. Although we asked people to answer ’yes’ only if they had been diagnosed by a physician at least once, we cannot completely rule out the possibility of misreporting on the questionnaire. It should also be mentioned that Type 1 and Type 2 diabetes were combined. Since the rates of Type 1 diabetes are rather low, this should not mean too much inaccuracy. It should also be noted that the prevalence of diabetes mellitus is based on self-reported data. Depending on the measurement (HbA1c, fasting plasma glucose, antidiabetic medication), there may be an over- or underestimation. For example, measurement of fasting plasma glucose alone may lead to underestimation. In comparison with other studies, a slight underestimation can possibly be assumed here.

It should also be added that there are various potential risk factors that could not be taken into account here due to their complexity. In addition to comorbidities, these also include dietary habits, occupational exposure, access to health care or environmental factors such as noise or air pollution. Further studies will attempt to take into account the risk factors not yet considered here.

## Conclusions

The results show that in specific regions within a large German city, people face a particularly increased disease burden from NCD. This in-depth small-scale regional study demonstrates the local needs for care and thus provides a starting point for health care planning and prevention of future health problems. These findings are relevant to Hamburg and beneficial for other large cities in Germany and beyond, where significant disparities in people’s living conditions may exist, resulting in unequal distribution of health outcomes.
